# Evaluation of the Contributions of Four Components of Gross Domestic Product in Various Regions in China

**DOI:** 10.1371/journal.pone.0121594

**Published:** 2015-04-27

**Authors:** Sanmang Wu, Yalin Lei, Li Li

**Affiliations:** 1 School of Humanities and Economic Management, China University of Geosciences, Beijing, 100083, China; 2 Key Laboratory of Carrying Capacity Assessment for Resource and Environment, Ministry of Land and Resource, Beijing, 100083, China; 3 School of Public Policy and Management, Tsinghua University, Beijing, 100084, China; Universidad Veracruzana, MEXICO

## Abstract

Four major components influence the growth of the gross domestic product in Chinese provinces: consumption, investment, transnational exports, and inter-provincial exports. By splitting a competitive input-output table into a non-competitive input-output table, this study used an input-output model to measure the contributions of the four components of gross domestic product in various regions in China. We found that international exports drove the growth of the gross domestic product more strongly in the eastern region than in other regions. Investment and inter-provincial exports were the major impetus for gross domestic product growth in the central and western regions. We also found that consumption played a minimal role in driving the growth of the gross domestic product in all regions in China. According to these findings, although various regions can share much in terms of policies to transform the impetus for economic growth, there should be different foci for different regions. Their shared policy is to increase the role of final consumption in stimulating economic growth. Region-specific policies mandate that the eastern region should strengthen the driving force provided by international exports and that the central and western regions should strengthen indigenous growth capabilities by improving scientific innovation, industrial support, and institutional innovation.

## Introduction

Since its reform and opening up to the outside world, China has made great strides in terms of economic and social development. Since 1978, China has maintained a 10% annual gross domestic product (GDP) growth rate. However, the problem of uneven, incompatible, and unsustainable economic and social development is still prominent. In terms of the driving forces for economic growth, China’s economic growth primarily depends on the pull of investment and exports and lacks the stimulation of consumption demand, which is particularly problematic [1].

Documents and directives from different levels of the Chinese government have continuously emphasized the need to support structural transformation in China. One of the most important implications of the transformation strategy is shifting the driving forces from exports and investment to include domestic consumption. Realizing such a transformation will require cooperation and coordination between the economic system and economic policy at a national level. However, there are 31 provinces (municipalities and autonomous regions), and they differ greatly in geographic conditions, natural resources, industrial structures, and developmental stages. For example, in 2013, the per capita GDP in Guangdong Province reached US$10,000, but the per capita GDP in Qinghai Province only reached US$6000. The driving forces of economic growth for one province are likely to be different from the driving forces for other provinces. To design regionally appropriate policies, it is crucial to understand the heterogeneous sources of growth for each province.

Three factors influence the growth of a country’s economy from a final demand perspective: consumption, investment, and international exports. For provinces, four factors influence economic growth from a demand perspective: consumption, investment, international exports, and inter-provincial exports [2]. Therefore, to gain a better understanding of the different sources of growth for different provinces, we aimed to measure the contributions of the four GDP components for each of the four main regions in China. China is generally divided into four regions: the eastern, central, western, and northeastern regions. The eastern region includes Beijing, Tianjin, Hebei, Shanghai, Jiangsu, Zhejiang, Fujian, Shandong, Guangdong, and Hainan provinces. The central region includes Shanxi, Anhui, Jiangxi, Henan, Hubei, and Hunan provinces. The western region includes Inner Mongolia, Guangxi, Chongqing, Sichuan, Guizhou, Tibet, Yunnan, Shann’xi, Gansu, Qinghai, Ningxia, and Xinjiang provinces. The northeastern region includes Liaoning, Jilin, and Heilongjiang provinces.

## Literature Review

According to the existing literature, there are three main types of quantitative research on the economic contribution of final demand.

The first type of quantitative research uses the identical equation of national revenue to decompose GDP into consumption, investment, and net exports and uses an elasticity formula to measure the contribution of final demand to economic growth. Typical examples of this type of research include studies by Wang [3] and Peng [4]. These authors found that exports had positive effects on China’s economic growth. Although this method makes data calculation simpler, it does not consider the relationship between imports, consumption, and investment and thus underestimates the contribution of foreign trade to economic growth. This method cannot be used to measure the contribution of exports. After comparing GDP data with net exports in previous years, Zhang and Hu[5] found that “net exports have ‘a negative correlation’ with GDP growth,” which also indicates that this method underestimates or inaccurately reflects the contribution of foreign trade.

The second type of quantitative research uses an econometric regression model to analyze the contribution of final demand to economic growth. Ghirmay’s study used time-series data for 15 low-income developing countries and a vector error correction model to examine the relationships among exports, investment, and economic growth [6]. Islam [7] used a vulnerability exploitation model to study the relationship between export expansion and economic growth in 15 Southeast Asian countries. Shan and Ken [8] used a multi-causality testing model to study the relationship between exports and China’s economic growth. Lin and Li [9] believed that to examine the relationship between exports and economic growth more comprehensively, the direct and indirect boosting effects of increased exports on economic growth must be considered. They used multiple measurement equations for estimation and found that since the 1990s, every 10% of foreign trade growth could result in 1% of GDP growth. Gao and Xu [10] used an econometric model to conduct an empirical analysis of the relationships among GDP, investment, consumption, and exports for Shandong Province. The results indicated that investment made the greatest contribution to GDP growth in Shandong Province, whereas exports made the smallest contribution. This method requires the use of time-series data over a long period, so it is more suitable for mature economies in which final demand makes a stable contribution to economic growth. However, for a country with rapid economic growth and changing structures (e.g., China), this method cannot accurately measure the contribution of final demand.

The third type of quantitative research involves using input-output (I-O) tables to measure the contribution of final demand to the economy. The I-O model that combines final demand and the production process is a suitable tool for measuring and calculating the pull effect of final demand. Chenery[11], Chenery et al. [12], Takahiro[13], Zakaria and Ahmad[14], and Tregenna[15–16] have conducted research regarding the application of this model. For example, Takahiro [13] used I-O data to analyze Indonesia’s sources of industrial growth between 1971 and 1985. Tregenna [15] used I-O data to analyze the sources of growth in South Africa from 1970 to 2007 and found that the dependence on domestic expansion has been a source of growth since 2000. Chinese scholars’ research to measure and calculate the economic contribution of final demand has primarily focused on the contribution of transnational exports. Lau et al. [17] constructed a non-competitive I-O model that can reflect the features of the Chinese processing trade. They also developed a methodology to calculate the pull effect of a country’s total transnational exports and unit exports for different sections and categories on the domestic value added and employment and indicated that the total transnational export value equals the sum of the absolute domestic value added and the amount of the imported products that is composed of exported products. Li et al. [18] measured the contribution of foreign trade to China’s economic growth and employment using a non-competitive I-O table. Shen’s (2009) groundbreaking paper used an I-O table to analyze the pull effect of demand on China’s economic growth [19]. The paper noted that the proportions of three demands that compose GDP by the expenditure approach (final consumption expenditure, gross capital formation, and net outflow of goods and services) cannot be viewed as the reasons for the pull effect of three components (consumption, investment, and transnational export) on GDP. The paper noted that the proportions of three demands that compose GDP through the expenditure approach (i.e., final consumption expenditure, gross capital formation, and net outflow of goods and services) could not explain the pull effect of three components (i.e., consumption, investment, and transnational exports) on GDP. Moreover, an I-O model calculated the pull effects of these three components on GDP by splitting a competitive I-O table into a non-competitive I-O table. The results indicated that the pull effect of consumption has decreased and the pull effect of transnational exports has increased since 2002.

The first method does not consider the relationship between imports, consumption, and investment and therefore underestimates the contribution of foreign trade to economic growth. The second method requires using time series data over a long period, so it is more suitable for mature economies in which final demand makes a stable contribution to economic growth; however, for a country with rapid economic growth and a changing structure, such as China, this method cannot accurately measure the contribution of final demand. We determined that the third type of quantitative research is most suitable for measuring the economic contribution of China’s final demand in various regions in China, especially the contribution in a given year. Therefore, this paper evaluates the contributions of the four GDP components in different regions using I-O tables.

## Materials and Methods

To evaluate the contributions of the four components of gross domestic product in various regions in China, first, this paper will construct non-competitive I-O tables by splitting the competitive I-O tables in the next part. Second, the paper will construct the measuring method with the contributions of four components of gross domestic product in various regions in China based on the input-output model.

### Basic model

According to the I-O model, the basic computational equation for the input and output of a region (province, region and city) is as follows:

$$X={\left(I-A\right)}^{-1}(Y-M-Z)$$ (1)

In Eq (1), X is the total output of one region, A is the direct input coefficient matrix, Y is the final demand matrix, M is the international import matrix, and Z is the inter-provincial import matrix. The matrices are obtained from each region’s competitive I-O tables (**Table 1**). A region’s competitive I-O table does not distinguish between intermediate inputs from imported products, inter-provincial imported products, and regional products. Therefore, the competitive I-O table cannot evaluate the contributions of the four GDP components. For this reason, regional products, international imported products, and inter-provincial imported products should be split apart to calculate a region’s non-competitive I-O table, as displayed in Table 2.

**Table 1:** Simplified Regional Competitive Input-Output Table for province.

**Projects**		**Intermediate demand** ^**a**^				**Final demand** ^**b**^					**International**	**Inter-provincial**	**Total**
	**sectors**	**1**	**2**	**…**	**n**	**Consumption**	**Capital formation**	**International exports** ^**e**^	**Inter-provincial exports** ^**f**^	**Total**	**imports** ^**c**^	**imports** ^**d**^	**outputs**
**Intermediate inputs**	**1**	*x*_11_	*x*_12_	**…**	*x*_1*n*_	*c*_1_	*in*_1_	*ex*_1_	*od*_1_	*Y*_1_	*M*_1_	*Z*_1_	*X*_1_
	**2**	*x*_21_	*x*_22_	**…**	x_2*n*_	*c*_2_	*in*_2_	*ex*_2_	*od*_2_	*Y*_2_	*M*_2_	*Z*_2_	*X*_2_
	**┋**	**┋**	**┋**	**…**	**┋**	**┋**	**┋**	**┋**	**┋**	**┋**	**┋**	**┋**	**┋**
	**n**	*x*_*n*1_	*x*_*n*2_	**…**	*x*_*nn*_	*c*_*n*_	*in*_*n*_	*ex*_*n*_	*od*_*n*_	$Y_{n}^{d}$	*M*_*n*_	*Z*_*n*_	*X*_*n*_
**Value added**		*V*_1_	*V*_2_	**…**	*V*_*n*_	N/A	N/A	N/A	N/A	N/A	N/A	N/A	N/A
**Total Inputs**		*X*_1_	*X*_2_	**…**	*X*_*n*_	N/A	N/A	N/A	N/A	N/A	N/A	N/A	N/A

^a^Intermediate demand is the consumption on the part of other industries (including the industry itself) for the production of a certain industry in the production process.

^b^Final demand is the end use or consumption of the whole society for a certain industry, and for provinces, final demand includes consumption, capital formation, international exports and inter-provincial.

^c^International imports for provinces are the products or services purchased from abroad.

^d^Inter-provincial imports are the products or services purchased from the rest of the country.

^e^International exports for provinces are the products or services sold abroad.

^f^Inter-provincial exports are the products or services sold to the rest of the country.

**Table 2:** Simplified Regional Non-competitive Input-Output Table for province.

Projects		Intermediate demand				Final demand					International	Inter-provincial	Total
	sectors	1	2	…	n	Consumption	Capital formation	International exports	Inter-provincial exports	Total	imports	imports	outputs
**Intermediate input of regional products** ^**a**^	**1**	$x_{11}^{d}$	$x_{12}^{d}$	**…**	$x_{1n}^{d}$	$c_{1}^{d}$	$in_{1}^{d}$	$ex_{1}^{d}$	$od_{1}^{d}$	$Y_{1}^{d}$	N/A	N/A	*X*_1_
	**2**	$x_{21}^{d}$	$x_{22}^{d}$	**…**	$x_{2n}^{d}$	$c_{2}^{d}$	$in_{2}^{d}$	$ex_{2}^{d}$	$od_{2}^{d}$	$Y_{2}^{d}$	N/A	N/A	*X*_2_
	**┋**	**┋**	**┋**	**…**	**┋**	**┋**	**┋**	**┋**	**┋**	**┋**	**┋**	**┋**	**┋**
	**N**	$x_{n1}^{d}$	$x_{n2}^{d}$	**…**	$x_{nn}^{d}$	$c_{n}^{d}$	$in_{n}^{d}$	$ex_{n}^{d}$	$od_{n}^{d}$	$Y_{n}^{d}$	N/A	N/A	*X*_*n*_
**Intermediate inputs of international imported products** ^**b**^	**1**	$x_{11}^{m}$	$x_{12}^{m}$	**…**	$x_{1n}^{m}$	$c_{1}^{m}$	$in_{1}^{m}$	$ex_{1}^{m}$	$od_{1}^{m}$	$Y_{1}^{m}$	*M*_1_	N/A	N/A
	**2**	$x_{21}^{m}$	$x_{22}^{m}$	**…**	$x_{2n}^{m}$	$c_{2}^{m}$	$in_{2}^{m}$	$ex_{2}^{m}$	$od_{2}^{m}$	$Y_{2}^{m}$	*M*_2_	N/A	N/A
	**┋**	**┋**	**┋**	**…**	**┋**	**┋**	**┋**	**┋**	**┋**	**┋**	**┋**		
	**n**	$x_{n1}^{m}$	$x_{n2}^{m}$	**…**	$x_{nn}^{m}$	$c_{n}^{m}$	$in_{n}^{m}$	$ex_{n}^{m}$	$od_{n}^{m}$	$Y_{n}^{m}$	*M*_n_	N/A	N/A
**Intermediate inputs of inter-provincial imported products** ^**c**^	**1**	$x_{11}^{z}$	$x_{12}^{z}$	**…**	$x_{1n}^{z}$	$c_{i}^{z}$	$in_{1}^{z}$	$ex_{1}^{z}$	$od_{1}^{z}$	$Y_{1}^{z}$	N/A	*Z*_1_	N/A
	**2**	$x_{21}^{z}$	$x_{22}^{z}$	**…**	$x_{2n}^{z}$	$c_{2}^{z}$	$in_{2}^{z}$	$ex_{2}^{z}$	$od_{2}^{z}$	$Y_{2}^{z}$	N/A	*Z*_2_	N/A
	**┋**	**┋**	**┋**	**…**	**┋**	**┋**	**┋**	**┋**	**┋**	**┋**		**┋**	
	**n**	$x_{n1}^{z}$	$x_{n2}^{z}$	**…**	$x_{nn}^{z}$	$c_{n}^{z}$	$in_{n}^{z}$	$ex_{n}^{z}$	$od_{n}^{z}$	$Y_{n}^{z}$	N/A	*Z*_*n*_	N/A
**Value added**		*V*_1_	*V*_2_	**…**	*V*_*n*_	N/A	N/A	N/A	N/A	N/A	N/A	N/A	N/A
**Total Inputs**		*X*_1_	*X*_2_	**…**	*X*_*n*_	N/A	N/A	N/A	N/A	N/A	N/A	N/A	N/A

^a^Intermediate inputs of regional products are the products that are used for intermediate inputs and are from the province itself.

^b^Intermediate inputs of international imported products are the products that are used for intermediate inputs and are from abroad.

^c^Intermediate inputs of inter-provincial imported products are the products that are used for intermediate inputs and are from the rest of the country.

In the following tables and equations, the superscript *d* refers to a region’s product, the superscript *m* refers to international imported products, and the superscript *z* refers to inter-provincial imported products. The lower case letters refer to flows, and the capital letters refer to totals.

### Equilibrium equations

The following equilibrium equations can be obtained according Table 2.

First, the equilibrium equation of a region’s product is as follows:

$$\sum_{j=1}^{n}x_{ij}^{d}+Y_{i}^{d}=X_{i}\;i=1,2,\cdots ,n$$ (2)

Suppose a region’s direct input coefficient isaijd=xijdXj, *j* = 1,2,⋯, *n*; introduce $a_{ij}^{d}$ into Eq (2), and the following equation can be obtained:

$$\sum_{j=1}^{n}a_{ij}^{d}X_{j}+Y_{i}^{d}=X_{i}\;i=1,2,\cdots ,n$$ (3)

Eq (3) can be written in matrix form, *A* ^*d*^ *X* + *Y* ^*d*^ = *X*, and Eq (4) can be obtained:

$$X={\left(I-A^{d}\right)}^{-1}Y^{d}$$ (4)

In Eq (4), (I − *A* ^*d*^)^−1^ = *B* ^*d*^ is a Leontief inverse matrix of a region’s product, where the element${\bar{b}}_{ij}^{d}$refers to the total demand on *i* goods per unit of sector *j*.

Second, for the international imported products in Table 2, the equilibrium equation is as follows:

$$\sum_{j=1}^{n}x_{ij}^{m}+Y_{i}^{m}=M_{i}\;i=1,2,\cdots ,n$$ (5)

Suppose the direct input coefficient of international imported products is$a_{ij}^{m}=\frac{x_{ij}^{m}}{X_{j}}$, *j* = 1,2,⋯, *n*; introduce $a_{ij}^{m}$ into Eq (5), and the following equation can be obtained:

$$\sum_{j=1}^{n}a_{ij}^{m}X_{j}+Y_{i}^{m}=M_{i}\;i=1,2,\cdots ,n$$ (6)

Eq (6) can also be written as a matrix formula:

$$A^{m}X+Y^{m}=M$$ (7)

In Eq (7), *A* ^*m*^ is a direct input coefficient matrix of international imported products.

Place X from Eq (4) into Eq (7) to obtain Eq (8) or Eq (9):

$$M=A^{m}{\left(I-A^{d}\right)}^{-1}Y^{d}+Y^{m}$$ (8)

$$M-Y^{m}=A^{m}{\left(I-A^{d}\right)}^{-1}Y^{d}$$ (9)

In Eq (9), *M* − *Y* ^*m*^ represents the imported products that are being used for intermediate inputs. *A* ^*m*^(*I* − *A* ^*d*^)^−1^ is still a matrix in which the two matrices having *n* order matrix multiply. This matrix is the *B* ^*m*^ complete input coefficient of the international imported products, wherein element *i* in rank *k* is the complete consumption (i.e., complete demand) of the domestic final products of sector *k* per unit to the international imported products of type *i*.

Third, for the inter-provincial imported products in Table 2, the equilibrium equation is as follows:

$$\sum_{j=1}^{n}x_{ij}^{z}+Y_{i}^{z}=Z_{i}$$ (10)

Suppose the direct input coefficient of the inter-provincial imported products is$a_{ij}^{z}=\frac{x_{ij}^{z}}{X_{j}}$, *j* = 1,2,⋯, *n*; introduce $a_{ij}^{z}$ into Eq (10), and the following equation can be obtained:

$$\sum_{j=1}^{n}a_{ij}^{z}X_{j}+Y_{i}^{z}=Z_{i}\;i=1,2,\cdots ,n$$ (11)

Eq (11) can be written in a matrix form as follows:

$$A^{z}X+Y^{z}=Z$$ (12)

In Eq (12), *A* ^*z*^ is the direct input coefficient matrix of the inter-provincial imported products.

$$A^{d}+A^{m}+A^{z}=A◦$$

Place X from Eq (4) into Eq (12) to obtain the following Eq (13) or Eq (14):

$$Z\;=\;A^{z}{\left(I-A^{d}\right)}^{-1}Y^{d}\;+\;Y^{z}$$ (13)

$$Z-Y^{z}\;=\;A^{z}{\left(I-A^{d}\right)}^{-1}Y^{d}$$ (14)

In Eq (14), *Z* − *Y* ^*m*^ represents the inter-provincial imported products that are being used for intermediate input. *A* ^*z*^(*I* − *A* ^*d*^)^−1^ is still a matrix in which the two *n*-order matrices are multiplied. This matrix is the *B* ^*z*^ complete input coefficient of the inter-provincial imported products, wherein element *i* in rank *k* is the complete consumption (i.e., complete demand) of the domestic final products of sector *k* per unit to the inter-provincial imported products of type *i*.

### Contributions of the four components to GDP

If the value added of the total production input of sector *j* per unit (i.e., the value-added rate) is $r_{j}=\frac{V_{j}}{X_{j}},$ then *V*_*j*_ = *r*_*j*_ • *X*_*j*_, *j* = 1,2,⋯, *n*. The sum of each sector’s value added is GDP:

$$GDP=\sum_{j=1}^{n}V_{j}=\sum_{j=1}^{n}r_{j}X_{j}=RX$$ (15)

In Eq (15), *R* = (*r*_1_, *r*_2_,⋯, *r*_*n*_) is the row vector of the value-added rate, and *X* is the column vector of the total input (i.e., total production). Placing Eq (4) into Eq (15) results in the following:

$$GDP=RX=R{\left(I-A^{d}\right)}^{-1}Y^{d}$$ (16)

Because *Y* ^*d*^ = *C* ^*d*^ + *IN* ^*d*^ + *EX* ^*d*^ + *OD* ^*d*^, placing *Y* ^*d*^ into Eq (16) results in the following equation:

$$\begin{array}{l} GDP=RX=R{\left(I-A^{d}\right)}^{-1}(C^{d}+IN^{d}+EX^{d}+OD^{d})\\ \;=R{\left(I-A^{d}\right)}^{-1}C^{d}\;+R{\left(I-A^{d}\right)}^{-1}IN^{d}\;+R{\left(I-A^{d}\right)}^{-1}EX^{d}\;+R{\left(I-A^{d}\right)}^{-1}OD^{d}\\ \;=GDP^{C}+GDP^{IN}+GDP^{EX}+GDP^{OD} \end{array}$$ (17)

In Eq (17), *GDP* ^*C*^, *GDP* ^*IN*^, *GDP* ^*EX*^, and *GDP* ^*OD*^ are GDP-driven by consumption *C* ^*d*^, investment *IN* ^*d*^, international export *EX* ^*d*^, and inter-provincial export *OD* ^*d*^, respectively.

The common left multiplication *R*(*I* − *A* ^*d*^)^−1^ that is shared by *GDP* ^*C*^, *GDP* ^*IN*^, *GDP* ^*EX*^, and *GDP* ^*OD*^ is a row vector that can be called vector *p*, where *p* = (*p*_1_.*p*_2_,⋯ *p*_*n*_) of the driving force of the final product, which is composed of GDP driven by each sector’s final product per unit. Element *j* corresponds to GDP by the final product of sector *j* per unit:

$$\begin{array}{l} p=(p_{1,}p_{2},\cdots ,p_{n})=R{\left(I-A^{d}\right)}^{-1}=(r_{1},r_{2},\cdots ,r_{n})\left[\begin{array}{cccc} b_{11}^{d} & b_{12}^{d} & \cdots & b_{1n}^{d}\\ b_{21}^{d} & b_{22}^{d} & \cdots & b_{2n}^{d}\\ \vdots & \vdots & \vdots & \vdots \\ b_{n1}^{d} & b_{n2}^{d} & \cdots & b_{nn}^{d} \end{array}\right]\\ =(\sum_{k=1}^{n}r_{k}b_{k1}^{d},\sum_{k=1}^{n}r_{k}b_{k2}^{d},\cdots ,\sum_{k=1}^{n}r_{k}b_{kn}^{d}) \end{array}$$ (18)

The proportion of GDP driven by a region’s consumption element is as follows:

$$\pi_{c}=\frac{GDP^{C}}{GDP}$$ (19)

The proportions of GDP driven by the other four elements can likely be obtained. It is easy to discover the following reasonable equation:

$$\pi_{c}+\pi_{In}+\pi_{Ex}+\pi_{OD}=1$$ (20)

According to Eq (17), if GDP and the data for each final product are converted into comparable prices and increment is considered, the following equation results:

$$\Delta GDP_{p}=\Delta GDP_{p}^{c}+\Delta GDP_{p}^{IN}+\Delta GDP_{p}^{EX}+\Delta GDP_{p}^{OD},$$ (21)

where Δ*GDP*_*p*_ is the increment of the total value of a region’s output that is calculated by comparable prices;$\Delta GDP_{P}^{C}$, $\Delta GDP_{P}^{IN}$, $\Delta GDP_{P}^{EX}$, and $\Delta GDP_{P}^{OD}$are the consumption expenditures, capital formation, international exports, and inter-provincial exports, respectively.

Divide Δ*GDP*_*p*_ in the two sides of Eq (21) to obtain the following equation:

$$1=\frac{\Delta GDP_{p}^{c}}{\Delta GDP_{p}}+\frac{\Delta GDP_{p}^{IN}}{\Delta GDP_{p}}+\frac{\Delta GDP_{p}^{EX}}{\Delta GDP_{p}}+\frac{\Delta GDP_{p}^{OD}}{\Delta GDP_{p}}$$ (22)

On the right side of Eq (22) are the contribution rates of the growth of a region’s four demands for the GDP of a region. The two sides of Eq (21) are multiplied by the GDP growth rate (i.e., percentage points) as follows:

$$g=\frac{\Delta GDP_{p}^{C}}{\Delta GDP_{p}}g+\frac{\Delta GDP_{p}^{IN}}{\Delta GDP_{p}}g+\frac{\Delta GDP_{p}^{EX}}{\Delta GDP_{p}}g+\frac{\Delta GDP_{p}^{OD}}{\Delta GDP_{p}}g$$ (23)

On the right side of Eq (23) are the percentage points of a region’s economic growth that is driven by that region’s four demands.

### Data Sources

Our first data source for inter-provincial exports, inter-provincial imports, international exports, and international imports was the official regional I-O tables of the National Bureau of Statistics of China (China’s National Bureau of Statistics)[20–22]. The 1997 data for each region’s inter-provincial exports, inter-provincial imports, international exports, and international imports per sector are explicit in the regional I-O tables. However, in 2002, the I-O tables of 13 provinces (regions and cities) were not as detailed. In 2007, none of the I-O tables was as detailed as those from 1997. To resolve these issues, we supplemented the I-O tables with customs data [23–25]. The data on international imports and exports in different industries were collected from customs according to different sources and different destinations. Next, we obtained the data for each province’s inter-provincial exports and inter-provincial imports by subtracting the international exports and imports from the total exports and total imports (in the table) for each province.

Our main contribution is the transformation of each province’s competitive I-O table into a non-competitive I-O table. As discussed above, the competitive I-O tables cannot be used to evaluate the contributions of the four GDP components, and they thus need to be split into non-competitive I-O tables. Because the trade items in each province’s I-O table are designated as inter-provincial exports, inter-provincial imports, international exports, and international imports, it is further supposed that the products from inside a region, abroad, and other provinces (regions and cities) are homogeneous [26]. To overcome the difficulty associated with data collection, Chenery [27] and Moses [28] have proposed the Chenery-Moses model. This model assumes that different goods (intermediate input, final consumption, and investment in different sectors) in each region are from the same source. For example, in terms of the source, two-thirds of the coal consumed by Beijing is assumed to come from Shanxi and one-third is assumed to come from Hebei. In reality, the proportion of coal consumed from the source by different sectors of Beijing may be different, but we assume that the proportion of coal consumed from the source by different sectors of Beijing is the same as the assumption. The following equations can thus be obtained:

$$y_{i}^{m}=M_{i}\frac{y_{i}}{\sum_{j=1}^{n}x_{ij}+\sum_{j=1}^{k}y_{j}}$$ (24)

$$x_{ij}^{m}=M_{i}\frac{x_{ij}}{\sum_{j=1}^{n}x_{ij}+\sum_{j=1}^{k}y_{j}}$$ (25)

$$y_{i}^{z}=Z_{i}\frac{y_{i}}{\sum_{j=1}^{n}x_{ij}+\sum_{j=1}^{k}y_{j}}$$ (26)

$$x_{ij}^{z}=Z_{i}\frac{x_{ij}}{\sum_{j=1}^{n}x_{ij}+\sum_{j=1}^{k}y_{j}}$$ (27)

where *y*_*i*_ refers to the final use amount of item *i* in the original competitive I-O table, and *x*_*ij*_ refers to the amount of sector *i* total consumption during the sector *j* production process in the original competitive I-O table.

Based on the four Eq (24–27) above, the non-competitive I-O tables can be obtained.

## Results and Analysis

### Contributions of the four components to GDP in various regions in China

**Fig 1:**
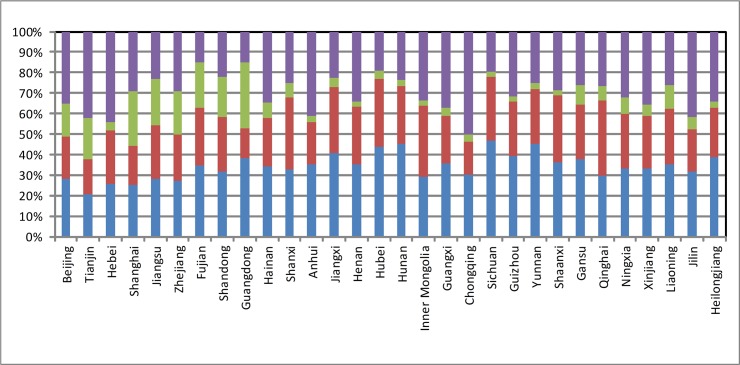
Contributions of the four GDP components in various regions in China (2007). For provinces, four factors influence economic growth from a demand perspective: consumption, investment, international exports, and inter-provincial exports. The purple represents the influence of inter-provincial exports; the green represents the influence of international exports, the red represents the influence of investment, the blue represents the influence of consumption in the Fig 1

As indicated in **Fig 1.**, remarkable heterogeneity existed in the driving factors of GDP in various regions in China in 2007.

Data for the contributions of the four components in different provinces use the I-O tables for the year 2007.

International exports contributed much more to GDP in the eastern region than in any other regions. In 2007, the top eight provinces and cities where international exports were the most prominent driver of GDP were Guangdong (32.1%), Shanghai (26.6%), Jiangsu (22.5%), Fujian (22.1%), Zhejiang (20.9%), Shandong (19.2%), Tianjin (19.7%), and Beijing (16.2%), all of which are located in the eastern region. In contrast, the bottom eight regions, where international exports drove GDP the least were Guizhou (2.2%), Hunan (2.7%), Shaanxi (2.74%), Henan (2.78%), Inner Mongolia (2.83%), Sichuan (2.86%), Heilongjiang (2.9%), and Yunnan (2.91%), all of which are in the central and western regions. Accordingly, the role of international exports in stimulating economic growth varied from region to region, with the strongest impetus in the east and relatively smaller impetus in the central, west, and northeastern regions.

There are several reasons for this difference. First, China has long pursued imbalanced development by prioritizing the growth of the eastern region (since the passage of the reform and opening-up policies, which favored the development of trade in the eastern region). Second, the eastern region has made a great effort to attract foreign investment with the strategy of intensifying export-oriented international subcontracting and has become a part of the global value chain (Liu et al.)[29]. The manufacturing sector, which includes particularly highly international export-oriented manufacturers, saw rapid growth. International exports have increasingly driven the eastern region’s economic growth. Finally, the hinterland regions were late in the opening-up initiative, face high costs for importing intermediate products of moderate value from coastal regions and abroad, and have a relatively poor investment environment and high operating costs, which results in the relatively slow development of manufacturing and the reliance on resource-based industries.

Inter-provincial exports play a stronger role in driving GDP in the central, western, and northeastern regions than in the eastern region. In 2007, the top seven regions for GDP-driving inter-provincial exports were the Chongqing (50.3%), Hebei (44.42%), Tianjin (42.4%), Jilin (41.6%), Anhui (41.1%), Guangxi (37.3%), and Xinjiang (35.5%) provinces, and the majority of these are western and northeastern provinces. After the reform and opening-up initiative, the eastern region of China was the first to embrace global production chains, especially intra-product specialization, given its advantageous geographic location and initial endowments. This early development in the eastern region created further comparative advantages, which resulted in specific industrial concentrations in particular areas and improved the eastern region’s ability to sustain rapid growth. However, the eastern region’s comparative advantage in joining global production chains relied on low-priced production factors, and the central and western regions have similar comparative advantages today.

As a result of the low-priced labor and natural materials transported to the East, China’s trade pattern was formed; cheap labor and natural resources flow from the central and western regions to the East, and the East provides low-value products to the world through international export-oriented processing trade. Specifically, China has two economic growth modes: one driven by international exports (represented by eastern provinces and cities, such as Guangdong, Zejiang, Jiangsu, and Fujian) and one driven by inter-provincial exports (represented by central and western provinces and regions, such as Shaanxi, Hebei, Tianjin, Inner Mongolia, Jilin, Heilongjiang, and Xinjiang).

The role of consumption in driving GDP was strong in the central, western, and northeastern regions but relatively weak in the East. In 2007, the top eight regions for GDP-driving consumption were Sichuan (46.6%), Yunnan (45.3%), Hunan (45.3%), Hubei (43.7%), Jiangxi (40.5%), Guizhou (39.4%), Hei Longjiang (38.8%), and Gansu (37.9%), all of which are located in the central and western regions. The bottom eight regions for GDP-driving consumption were Tianjin (20.6%), Shanghai (25.2%), Hebei (25.5%), Jejiang (27.3%), Jiangsu (27.9%), Beijing (28.3%), Inner Mongolia (28.8%), and Qinghai (29.6%), which are mainly in the eastern regions.

Capital formation served as a stronger GDP-driving impetus in the central and western provinces (cities and regions) than in other regions. In 2007, the top eight regions for GDP-driving capital formation were Qinghai (36.6%), Shanxi (35.2%), Inner Mongolia (34.7%), Hubei (32.9%), Shaanxi (32.7%), Jiangxi (32.2%), Sichuan (31.1%), Fujian (28.4%), Hunan (28.4%), and Henan (28.1%), which are located mainly in the central and western regions.

### Changing contribution patterns for the four GDP components in various regions in China

Although the previous discussion elaborates on the roles of the four components in driving GDP in various Chinese regions, this analysis is static. Therefore, we also investigated detailed data from 1997, 2002, and 2007. As indicated in Table 3, from 1997 to 2007, the characteristics of the contributions of the four components to GDP in various regions in China were as follows:

**Table 3:** Contributions of the four GDP components in various regions in China in 1997, 2002, and 2007.

Regions		Consumption			Capital formation			International exports			Inter-provincial exports		
		1997	2002	2007	1997	2002	2007	1997	2002	2007	1997	2002	2007
Eastern region	Beijing	32.1	33.3	28.29	23.2	17.8	20.34	14.8	8.5	16.17	29.9	40.3	35.20
	Tianjin	23.1	25.3	20.63	18.3	15.9	17.29	14	17.9	19.66	44.6	40.9	42.42
	Hebei	26.3	31.6	25.48	23	21.7	26.16	3.5	3.2	3.95	47.2	43.5	44.42
	Shanghai	21.0	32.3	25.16	24.2	17.1	19.17	20	22.6	26.55	34.9	28	29.11
	Jiangsu	32.7	36.8	27.91	21.9	27.5	26.58	9.8	16.3	22.47	35.5	19.4	23.04
	Zhejiang	31.6	31.0	27.30	28.8	20.6	22.49	14.3	15.7	20.92	25.3	32.7	29.29
	Fujian	31.9	34.9	34.57	26.3	23.3	28.43	22.1	22.1	22.07	19.7	19.7	14.93
	Shandong	37.6	36.7	31.57	23.3	27.6	26.98	10.3	10.0	19.20	28.9	25.8	22.25
	Guangdong	30.4	44.9	37.96	16.4	14	15.05	33	27.7	32.10	20.2	13.4	14.88
	Hainan	36.4	39.0	34.27	20.9	19.6	23.50	8.4	4.0	7.33	34.3	37.4	34.90
	Weighted average	31.0	36.2	30.24	22.5	21.1	22.55	16.3	16.8	21.73	30.2	26.0	25.47
Central region	Shanxi	32.8	41.3	32.78	17.6	27.9	35.16	7.9	6.8	6.80	41.6	24	25.26
	Anhui	34.3	38.4	35.30	19.3	18.6	20.47	3.2	2	3.13	43.1	40.9	41.09
	Jiangxi	45.7	48.9	40.48	21.3	24.9	32.24	3.5	1.8	4.72	29.4	24.4	22.57
	Henan	41.8	48.9	35.27	27.3	29.3	28.02	2.8	2.2	2.78	28.1	19.6	33.93
	Hubei	44.5	45.7	43.71	24.3	31.9	32.97	4.1	2.6	4.48	27.1	19.8	18.84
	Hunan	48.2	52.6	45.21	20.5	23.9	28.38	3	2.1	2.74	28.4	21.3	23.67
	Weighted average	41.91	46.58	38.80	22.62	26.52	29.13	3.71	2.65	3.77	31.74	24.22	28.30
Western region	Inner Mongolia	38.7	35.9	28.92	17.9	29.8	34.70	3.4	2.4	2.83	40.1	31.8	33.55
	Guangxi	35.5	38.5	35.46	23.5	20.5	23.54	3.7	3.7	3.67	37.3	37.3	37.33
	Chongqing	37.4	31.9	29.96	16.6	13.1	16.09	2.4	1.9	3.60	43.6	53.1	50.34
	Sichuan	53.6	54.2	46.63	23.3	27.7	31.06	3.7	2.7	2.86	19.4	15.5	19.45
	Guizhou	54.2	44.4	39.36	12.2	22.2	26.69	3.3	2.3	2.23	30.4	31.1	31.72
	Yunnan	44.6	53.6	45.27	24.4	23.9	26.78	4.8	2.2	2.91	26.3	20.3	25.04
	Shaanxi	41.7	42.9	35.95	22.2	28.2	32.71	4.8	0.1	2.74	31.4	28.8	28.59
	Gansu	46.3	48.5	37.92	20.6	25.7	26.66	2.3	5.3	9.43	30.8	20.5	25.99
	Qinghai	51.7	34.5	29.62	-5.4	32.2	36.62	3.2	4.5	7.05	50.5	28.8	26.72
	Ningxia	37.3	43.9	33.30	23.9	29.2	26.61	4.1	1.3	7.75	34.7	25.6	32.34
	Xinjiang	40.2	40.8	33.19	19.5	23.4	25.86	2.5	3.2	5.47	37.7	32.5	35.48
	Weighted average	44.13	44.58	37.65	21.02	24.54	28.19	3.60	2.55	3.71	31.28	28.34	30.45
Northeastern region	Liaoning	38.8	40	35.14	22.7	21.8	27.21	11.9	10.3	11.48	26.6	28	26.17
	Jilin	41	36.5	31.74	18.3	16.8	20.46	5.5	2.8	6.18	35.2	43.9	41.62
	Hei Longjiang	37.7	43.9	38.89	17.3	18.2	23.81	6.3	2.5	2.91	38.7	35.3	34.39
	Weighted average	38.83	40.52	35.49	19.95	19.63	24.65	8.70	6.28	7.71	32.52	33.58	32.15

Data source: Authors’ analysis of provincial I-O tables for the years 1997, 2002, and 2007.

First, the GDP-driving role of international exports in the eastern region was stronger than in other regions. In 1997 and 2002, the weighted average of the proportion of GDP driven by international exports in the eastern region was 16.3% and 16.8%, respectively, compared with 3.71% and 2.65% in the central region, 3.60% and 2.55% in the western region, and 8.70% and 6.28% in the northeastern region. Our findings were similar for specific provinces (regions and cities), which indicates that China’s eastern region is heavily dependent on international exports; however, the other regions’ dependencies on international exports are relatively low.

The GDP-driving role of inter-provincial exports in the central and western regions was generally higher than in the eastern region for each year studied. In 1997 and 2002, the weighted average of the proportion of GDP driven by inter-provincial exports in the central region was 31.74% and 24.2%, respectively, and 31.28% and 28.34% in the western region, 32.5% and 33.58% in the northeastern region, and 30.2% and 26.0% in the eastern region. Our findings were again similar for specific provinces (regions and cities), and we concluded that the role of inter-provincial exports is stronger in the central and western regions than in the eastern region.

The GDP-driving role of international exports in various regions in China has been increasing. The weighted average of the proportion of GDP driven by international exports in the eastern region grew from 16.8% in 2002 to 21.7% in 2007. The proportion of GDP driven by international exports grew from 2.65% in 2002 to 3.8% in 2007 in the central region, from 2.55% to 3.7% in the western region, and from 6.28% to 7.7% in the northeastern region.

Consumption’s role in driving economic growth is weakening. The weighted average of the proportion of GDP driven by consumption fell from 36.2% in 2002 to 30.24% in 2007 in the eastern region, from 46.58% to 38.3% in the central region, from 44.58% to 37.6% in the western region, and from 40.5% to 35.5% in the northeastern region.

### Contribution rates and percentages of the four components in driving economic growth in various regions in China

To calculate the contribution rates and percentages of the four GDP components that that drive economic growth, we applied the GDP deflator to deflate GDP and the GDP driven by these four components. We analyzed the data according to Eq (22) and (23). As presented in Table 4 and Table 5, economic growth in the central and western regions was much faster than the growth in the eastern region. Between 2002 and 2007, economic growth averaged 84% in the central region, 85% in the western region, and 82% in the eastern region. Based on the contribution rates and percentages of the four GDP components that drive economic growth, our findings are described below.

**Table 4:** Contributions of the four GDP components to economic growth (1997–2002).

Regions	GDP growth rates	Percentage points of growth driven by the four GDP components				% contribution of the four GDP components			
	%	Consumption	Capital formation	International exports	Inter-provincial exports	Consumption	Capital formation	International exports	Inter-provincial exports
Beijing	0.69	0.24	0.07	0.00	0.38	0.24	**0.07**	**0.00**	0.38
Tianjin	0.68	0.19	0.08	0.16	0.24	0.19	0.08	0.16	0.24
Hebei	0.58	0.23	0.11	0.02	0.21	0.23	0.11	0.02	0.21
Shanghai	0.66	0.33	0.04	0.18	0.12	0.33	0.04	0.18	0.12
Jiangsu	0.66	0.29	0.24	0.17	-0.03	0.29	0.24	0.17	-0.03
Zhejiang	0.68	0.20	0.06	0.12	0.29	0.20	0.06	0.12	0.29
Fujian	0.59	0.24	0.11	0.13	0.12	0.24	0.11	0.13	0.12
Shandong	0.65	0.23	0.22	0.06	0.14	0.23	0.22	0.06	0.14
Guangdong	0.69	0.45	0.07	0.14	0.02	0.45	0.07	0.14	0.02
Hainan	0.53	0.23	0.09	-0.02	0.23	0.23	0.09	-0.02	0.23
Weighted Average	**0.64**	**0.26**	**0.11**	**0.10**	**0.17**	**0.26**	**0.11**	**0.10**	**0.17**
Shanxi	0.60	0.33	0.27	0.03	-0.03	0.33	0.27	0.03	-0.03
Anhui	0.53	0.24	0.09	0.00	0.19	0.24	0.09	0.00	0.19
Jiangxi	0.50	0.28	0.16	-0.01	0.07	0.28	0.16	-0.01	0.07
Henan	0.54	0.33	0.18	0.01	0.02	0.33	0.18	0.01	0.02
Hubei	0.51	0.25	0.24	0.00	0.03	0.25	0.24	0.00	0.03
Hunan	0.52	0.32	0.16	0.00	0.04	0.32	0.16	0.00	0.04
Weighted Average	**0.53**	**0.29**	**0.18**	**0.00**	**0.05**	**0.29**	**0.18**	**0.00**	**0.05**
Inner Mongolia	0.67	0.21	0.32	0.01	0.13	0.21	0.32	0.01	0.13
Guangxi	0.54	0.24	0.08	0.02	0.20	0.24	0.08	0.02	0.20
Chongqing	0.54	0.12	0.04	0.01	0.38	0.12	0.04	0.01	0.38
Sichuan	0.53	0.29	0.19	0.00	0.04	0.29	0.19	0.00	0.04
Guizhou	0.52	0.13	0.22	0.00	0.17	0.13	0.22	0.00	0.17
Yunnan	0.45	0.33	0.10	-0.02	0.03	0.33	0.10	-0.02	0.03
Shaanxi	0.66	0.29	0.25	-0.05	0.16	0.29	0.25	-0.05	0.16
Gansu	0.58	0.30	0.20	0.06	0.02	0.30	0.20	0.06	0.02
Qinghai	0.61	0.04	0.57	0.04	-0.04	0.04	0.57	0.04	-0.04
Ningxia	0.59	0.32	0.22	-0.02	0.06	0.32	0.22	-0.02	0.06
Xinjiang	0.48	0.20	0.15	0.02	0.10	0.20	0.15	0.02	0.10
Weighted Average	**0.56**	**0.23**	**0.21**	**0.01**	**0.11**	**0.23**	**0.21**	**0.01**	**0.11**
Liaoning	0.53	0.23	0.11	0.04	0.16	0.23	0.11	0.04	0.16
Jilin	0.54	0.15	0.08	-0.01	0.33	0.15	0.08	-0.01	0.33
Hei Longjiang	0.52	0.29	0.10	-0.03	0.15	0.29	0.10	-0.03	0.15
Weighted Average	**0.53**	**0.22**	**0.10**	**0.00**	**0.21**	**0.22**	**0.10**	**0.00**	**0.21**

Data source: Authors’ analysis of provincial I-O tables for the years 1997and 2002.

**Table 5:** Contributions of the four GDP components to economic growth (2002–2007).

Regions	GDP growth rates	Percentage points of growth driven by the four GDP components				% contribution of the four GDP components			
	%	Consumption	Capital formation	International exports	Inter-provincialExports	Consumption	Capital formation	International exports	Inter-provincial exports
Beijing	0.84	0.19	0.20	0.21	0.24	0.22	0.23	0.25	0.29
Tianjin	1.02	0.16	0.19	0.22	0.45	0.16	0.19	0.21	0.44
Hebei	0.83	0.15	0.26	0.04	0.38	0.18	0.32	0.05	0.46
Shanghai	0.85	0.14	0.18	0.27	0.26	0.17	0.22	0.31	0.3
Jiangsu	0.97	0.18	0.25	0.28	0.26	0.19	0.26	0.29	0.27
Zhejiang	0.94	0.22	0.23	0.25	0.24	0.23	0.25	0.27	0.26
Fujian	0.84	0.29	0.29	0.19	0.08	0.34	0.35	0.22	0.09
Shandong	0.97	0.25	0.26	0.28	0.18	0.26	0.26	0.29	0.19
Guangdong	0.98	0.30	0.16	0.36	0.16	0.31	0.16	0.37	0.16
Hainan	0.77	0.22	0.22	0.09	0.24	0.28	0.29	0.12	0.32
Weighted Average	**0.82**	**0.19**	**0.20**	**0.20**	**0.23**	**0.21**	**0.23**	**0.22**	**0.25**
Shanxi	0.96	0.23	0.41	0.07	0.26	0.24	0.43	0.07	0.27
Anhui	0.77	0.24	0.18	0.04	0.32	0.31	0.23	0.05	0.41
Jiangxi	0.83	0.25	0.34	0.07	0.17	0.3	0.41	0.08	0.2
Henan	0.88	0.18	0.24	0.03	0.44	0.2	0.27	0.03	0.5
Hubei	0.77	0.32	0.27	0.05	0.14	0.41	0.34	0.07	0.18
Hunan	0.79	0.28	0.27	0.03	0.21	0.36	0.34	0.04	0.27
Weighted Average	**0.84**	**0.25**	**0.28**	**0.05**	**0.26**	**0.3**	**0.34**	**0.06**	**0.3**
Inner Mongolia	1.50	0.36	0.57	0.05	0.52	0.24	0.38	0.03	0.35
Guangxi	0.82	0.26	0.22	0.03	0.31	0.32	0.27	0.04	0.37
Chongqing	0.83	0.23	0.16	0.05	0.39	0.28	0.2	0.06	0.47
Sichuan	0.84	0.31	0.29	0.03	0.20	0.38	0.35	0.03	0.24
Guizhou	0.79	0.26	0.26	0.02	0.26	0.33	0.32	0.02	0.33
Yunnan	0.65	0.21	0.20	0.03	0.21	0.32	0.31	0.04	0.32
Shaanxi	0.89	0.25	0.34	0.05	0.25	0.28	0.38	0.06	0.28
Gansu	0.73	0.17	0.20	0.11	0.24	0.23	0.28	0.15	0.34
Qinghai	0.81	0.19	0.34	0.08	0.20	0.24	0.42	0.1	0.24
Ningxia	0.77	0.15	0.18	0.12	0.31	0.19	0.23	0.16	0.41
Xinjiang	0.71	0.16	0.21	0.06	0.28	0.22	0.29	0.09	0.4
Weighted Average	**0.85**	**0.23**	**0.27**	**0.06**	**0.29**	**0.28**	**0.31**	**0.07**	**0.34**
Liaoning	0.86	0.25	0.29	0.11	0.21	0.29	0.33	0.13	0.24
Jilin	0.85	0.22	0.21	0.09	0.33	0.26	0.25	0.1	0.39
Heilongjiang	0.72	0.23	0.23	0.03	0.24	0.32	0.32	0.03	0.33
Weighted Average	**0.81**	**0.24**	**0.24**	**0.07**	**0.26**	**0.29**	**0.3**	**0.09**	**0.32**

Data source: Authors’ analysis of provincial I-O tables for the years 2002 and 2007.

The contribution rate of consumption to economic growth decreased. Between 1997 and 2002, economic growth averaged 64% in the eastern region, and consumption drove economic growth in the eastern region by 26 percentage points, with a contribution rate of 41%. Between 2002 and 2007, economic growth averaged 82% in the eastern region, and consumption drove economic growth in the eastern region by 19 percentage points, with a contribution rate of 21%. The situations in the western, central, and northeastern regions were similar.

The international export contribution rate to economic growth increased. Between 1997 and 2002, the international export contribution rate to economic growth was 14% in the eastern region, 1% in the central region, 1% in the western region, and nearly 0% in the northeastern region. However, between 2002 and 2007, the international export contribution rate to economic growth was 22% in the eastern region, 6% in the central region, 6% in the western region, and 9% in the northeastern region.

Investment and inter-provincial exports were clearly the major powerhouses for economic growth in the central, western, and northeastern regions. Between 2002 and 2007, inter-provincial exports drove economic growth in the eastern region by 23 percentage points, with a contribution rate of 25%; in the central region, by 26 percentage points, with a contribution rate of 34%; in the western region, by 29 percentage points, with a contribution rate of 25%; and in the northeastern region, by 26 percentage points, with a contribution rate of 32%. Despite the rapid economic growth in the central and western regions, the major impetus for growth was investment and resource output.

## Discussion and Conclusions

Based on the I-O tables of 30 provinces in 1997, 2002, and 2007, we constructed non-competitive I-O tables by splitting the competitive I-O tables. The effects of the four GDP components (i.e., consumption, investment, international exports, and inter-provincial exports) were evaluated in terms of driving economic growth in various regions in China. Our research results and conclusions are described below.

International exports drove economic growth more strongly in the eastern region than in other regions. In 2007, GDP driven by international exports in the eastern region (including the Guangdong, Shanghai, Jiangsu, Fujian, Zhejiang, and Shandong provinces) accounted for more than 20% of the total regional GDP. In comparison, GDP driven by international exports in the central and western regions (including Guizhou, Hunan, Shaanxi, Henan, and Inner Mongolia) was less than 2%.

Investment and inter-provincial exports were the major impetuses for economic growth in the central and western regions. In 2007, the proportion of GDP driven by inter-provincial exports of the overall GDPs in Chongqing, Jilin, Anhui, Guangxi, and Xinjiang was more than 35%.

Consumption played a minimal role in driving economic growth in all regions. In particular, in the eastern region, the proportion of GDP growth driven by consumption and the consumption contribution rate to economic growth were both low.

Given these conclusions, although various regions can share much in terms of policies to transform the impetus for economic growth, there should be different foci for different regions.

First, international exports drove economic growth more strongly in the eastern region than in the other regions. Thus, one of the most important implications of the transformation strategy is to shift driving forces from international exports to include domestic consumption, investment and inter-provincial exports. The eastern region should strengthen the driving force provided by international exports, which would require the eastern region to upgrade its manufacturing center by extending both ends of the industrial chain’s “smiling curve” to gain an advantageous position in the global value chain. The eastern region should strengthen the driving force provided by inter-provincial export, which would require the domestic value chain to be built on this advantageous position in the global value chain to form a development pattern that features the eastern region driving coordinated development in the central and western regions. The eastern region should improve the social security system and adjust the initial distribution mode of the national economy to spur consumer demand. This should help eliminate the inefficient growth model, which is dependent on international exports, and form a coordinated impetus mix featuring coordinated consumption, investment, international exports, and inter-provincial exports in the eastern region

Second, investment and inter-provincial exports were the major impetus for economic growth in the central and western regions. Thus, one of the most important implications of the transformation strategy is to shift driving forces from investment and inter-provincial exports to include domestic consumption and international exports. For the central and western regions, the most important issue is to strengthen indigenous growth capabilities by improving scientific innovation, industrial support, and institutional innovation. This focus should help eliminate the inefficient growth model, which is dependent on investment, and form a coordinated impetus mix that features coordinated consumption, investment, international exports, and inter-provincial exports.
